# Synergistic effects of contaminants in Lombardy waters

**DOI:** 10.1038/s41598-021-93321-6

**Published:** 2021-07-06

**Authors:** Caterina A. M. La Porta, Maria Rita Fumagalli, Stefano Gomarasca, Maria Chiara Lionetti, Stefano Zapperi, Stefano Bocchi

**Affiliations:** 1 Center for Complexity and Biosystems, University of Milan, via Celoria 16, 20133 Milan, Italy; 2 Department of Environmental Science and Policy, University of Milan, via Celoria 26, 20133 Milan, Italy; 3 CNR-Consiglio Nazionale delle Ricerche, Istituto di Biofisica, Via De Marini 6, 16149 Genoa, Italy; 4 Department of Physics, University of Milan, Via Celoria 16, 20133 Milan, Italy; 5 CNR-Consiglio Nazionale delle Ricerche, Istituto di Chimica della Materia Condensata e di Tecnologie per l’Energia, Via R. Cozzi 53, 20125 Milan, Italy

**Keywords:** Environmental impact, Computational biology and bioinformatics, Environmental sciences

## Abstract

Quantifying synergistic environmental effects in water contamination is still an open issue. Here, we have analyzed geolocalized data of pollutants recorded in 2018 in surface and groundwater of Lombardy, one of the areas with the highest agricultural production rates, not only in Italy, but also in Europe. Both herbicides and insecticides are present at concentration levels above the legal limit, mainly in surface waters. Geolocalized analysis allows us to identify interesting areas particularly affected by a combination of multiple pesticides. We thus investigated possible synergistic effects of these compounds on the environment, using the alga *C. reinhardtii* as a biosensor. Our results show that exposure for 7 days to four compounds, that we found present together at high concentration in surface waters, was able to induce a stress in the algae, as indicated by the presence of palmelloids. Our work results in a pipeline that could easily be exported to monitor other territories in Italy and abroad.

## Introduction

The increasing intensification in cropping system management carried out during the last decades led to a massive use of agrochemicals, particularly pesticides. Their intensive use is not always accompanied by increased yield, leading to important negative consequences for the environment and for the economic balance of the farm. In fact, on the one side, the massive use of agrochemicals creates the conditions for soil and water contamination and, on the other hand, it represents a high economic costs in terms of product purchase, distribution and management for the farm^1^. In October 2009, the European Union published the directive 128/2009 which establishes a framework to achieve a sustainable use of pesticides. Nowadays, the broad category of products used for plant/crop protection from stresses (Plant protection products PPP) is mainly regulated by framework Regulation (EC No 1107/2009).

Evidence that some of these chemical substances pose a potential risk to humans and other life forms and unwanted side effects to the environment is not new^2, 3^. In the last years, water contamination is becoming an increasingly serious problem worldwide and the regulatory jurisdiction specifies the maximum concentration level (MCL) that may occur for each pesticide as well as the impact on human health of the maximum concentration pesticide levels^4^. During the crop management cycle, farmers usually apply different pesticides resulting in possible multiple contamination of the environment, including water. Inside the broad category of pesticides, herbicides are the most extensively used, accounting for up to 50% of the global plant protection market^5^.

Even if a single pesticide could be at the same time effective and safe at a sufficiently low concentration, nowadays the main question is the impact of the accumulation of multiple pesticides in the environment, considering the entire ecosystem and including humans. The presence of chemical mixtures existing in samples of groundwater used for public supply was recently investigated by Toccalino and coworkers^6^. In that study, the authors showed that the combined exposure to different contaminants can be a potential concern for more than half of the samples of water studied and that, even though the water devoted to public supply is treated to reduce contamination according to the current legislation, it can still contain mixtures of the chemicals at worryingly high concentrations^6^.

Herein our study intends to create a pipeline for the geolocalization of water contaminants using a *big data* approach to identify the most abundant compounds present in waters, their co-presence and the hotspot sites where they are found. This pipeline allows us to monitor the territories and helps possible interventions to correct possible contamination. We focus our studies on the Lombardy region since here the agrofood sector is characterized by a very intensive model with one of the largest use of agrochemicals in Italy^7^. Moreover, Lombardyhas an interesting hydrography with many lakes, rivers, channels, and springs. In Lombardy there is also a high population density with about 420 people/km^2^, representing more than one-sixth of the entire Italian population.

In addition to the geolocalized statistical analysis, we investigated possible synergistic effects of multiple compounds, detected at concentration above the legal limit, using a biosensor, the alga *Chlamydomonas reinhardtii* (*C. reinhardtii*). Algae and microalgae are well known as biological indicators for the evaluation of the water and soil quality. In fact, they quickly respond to changes of the environmental quality allowing us to monitor synergistic and antagonistic effects of various pollutants^8^. Moreover, it is known that algae respond to environmental stress through phenotypic plasticity by forming reversible colonies, defined as palmelloids^9–11^.

## Materials and methods

### Data

Data relating pollutants in Lombardy in the year 2018 were provided by Regional Environment Protection Agency (ARPA). Lombardy ARPA monitors 492 groundwater stations and 350 points placed along the main river network monthly (temporary and small water bodies, often linked to the irrigation system, are excluded). The list of pollutants reported in the database are those provided by the European and Italian Water Directives (Dir. 2000/60/EC, Dir. 2006/118/EC, Dir. 2008/105/EC, Dir. 2009/90/EC, M.D. 56/2009, M.D. 260/2010, Leg. D. 15,/2006, Leg. D. 30/2009, Leg. D. 219/2010). Databases contain 160,160 and 269,858 records for groundwater and surface waters, respectively. We started from a total of 304 parameters for groundwater and of 435 for surface waters. These pollutants were subdivided into two subgroups: the first relating to herbicides and the second to insecticides. We identified 48 insecticides and 51 herbicides for groundwater and 48 insecticides and 51 herbicides for surface water (Table S1). In the data analysis, only the parameters with a concentration of $c > c_0=0.1$ μg/l were considered since this value is reported to be the legal limit value detected.

### Data analysis

Data were analyzed using a custom made python 3.7 jupyter notebook using the pandas package. Cluster of substances where obtained using the Apriori algorithm^12^ as implemented in the mlxtend package^13^. Apriori is an algorithm to determine association rules in databases. It is based on the identification of frequent individual items in the database and extending them to item sets that appear frequently together in the database. In the context of our analysis, the Apriori algorithm is used to find clusters of substances that were found together in the same location. We quantify the distribution of cluster sizes and then restrict our analysis to the most abundant clusters containing 2, 3 and 4 substances.

### Data visualization

Geolocalized shape files, rivers and location of land use and cover classes (DUSAF 2018) were obtained from the geoportal of the Lombardy region (http://www.geoportale.regione.lombardia.it) and plotted using python3.7 (https://www.python.org/) with the geopandas (0.6.1 https://geopandas.org), matplotlib (3.1.1 https://matplotlib.org/) and seaborn (0.11.0 https://seaborn.pydata.org/) packages. The rivers displayed in the maps are only those classified as “Reticolo idrico principale (RIP)”. Boxplots, barplots and heatmaps were plotted using the seaborn package.

### Chlamydomonas culture growth and exposure to stress conditions

*C. reinhardtii* cells were growth in TAP medium (cod. A1379801, Invitrogen) as batch cultures until they reached a concentration of $\approx 10^6$ cells/ml (corresponding to mid-exponential phase of growth). The cells were cultured under continuous cool-white fluorescent lamps ($\approx$ 100 μmol photons $\text{m}^{-2} \text{s}^{-1}$) within a 110rpm shaking incubator, at 25 °C^10^. 5ml of cells were spun at 1100 g/5 min/25 °C and resuspended in 15 ml of fresh TAP medium containing Glyphosate (10.3 μg/L, cod. 45521, Sigma-Merk), (aminomethyl)phosphonic acid (AMPA) (37.2 μg/L, cod. 324817, Sigma-Aldrich), Terbuthylazine (3.6 μg/L, cod. 45678, Merk-Millipore), Bentazon ( 6.4 μg/L, cod. 2052, Sigma-Aldrich) separately or in combination at the same concentrations detected in shallow water in 2018. Cells grown in TAP medium without contaminants have been used as control.

### Chlamydomonas growth and palmelloids quantification

The growth of *C. reinhardtii* was monitored by measuring optical density at 680 nm of 100 μL cell culture using a microplate reader (Ensight, Perkin Elmer), immediately after the seeding the cells and after the exposure to the compounds, alone or in combination^10^.

Palmelloids quantification. For each experimental condition, 100 μL of the cultured cells was harvested, washed in 1× Phosphate buffered saline (PBS, pH 7.0, cod. P4417, Sigma), and fixed overnight at 4 °C in 1% paraformaldehyde in PBS. The next day, cells were washed again, resuspended in 200 μL of 1× PBS and spotted onto a coverslip and images were acquired with DMi8 (Leica) using brightfield objective at 20×. To quantify the presence of palmelloids a custom pipeline including Trainable Weka Segmentation (TWEKA) Fiji plugin^14, 15^ and ICY (V. 1.9.5^16^) has been used for image pre-processing to optimize background recognition and area calculation. At least 500 cells or aggregates were considered for each condition.

The average size of single cells was observed to be $\approx 50\,\upmu \text{m}^2$, while smaller particles are probably due to debris or image segmentation artifacts. Similarly, very large aggregates are likely to represent either conglomerates or segmentation errors. Particles with size below 30 μm^2^ or above 250 μm^2^ were manually discarded following these observations. This choice does not affect the shape of distribution size presented in figure, but was only used to correctly count the total number of considered particles for normalization. Moreover, discarded data represent at most $\approx$ 5% of the data.

## Results

We investigated the quantity of herbicides and insecticides in surface waters and groundwater recorded by ARPA in Lombardy in the year 2018. Herbicides and insecticides represent 33% of the total parameters analyzed for surface waters and 36% for groundwater (see Table S2). The dataset includes a total of 160,160 records for groundwater and 269,858 records for surface waters.

**Figure 1:**
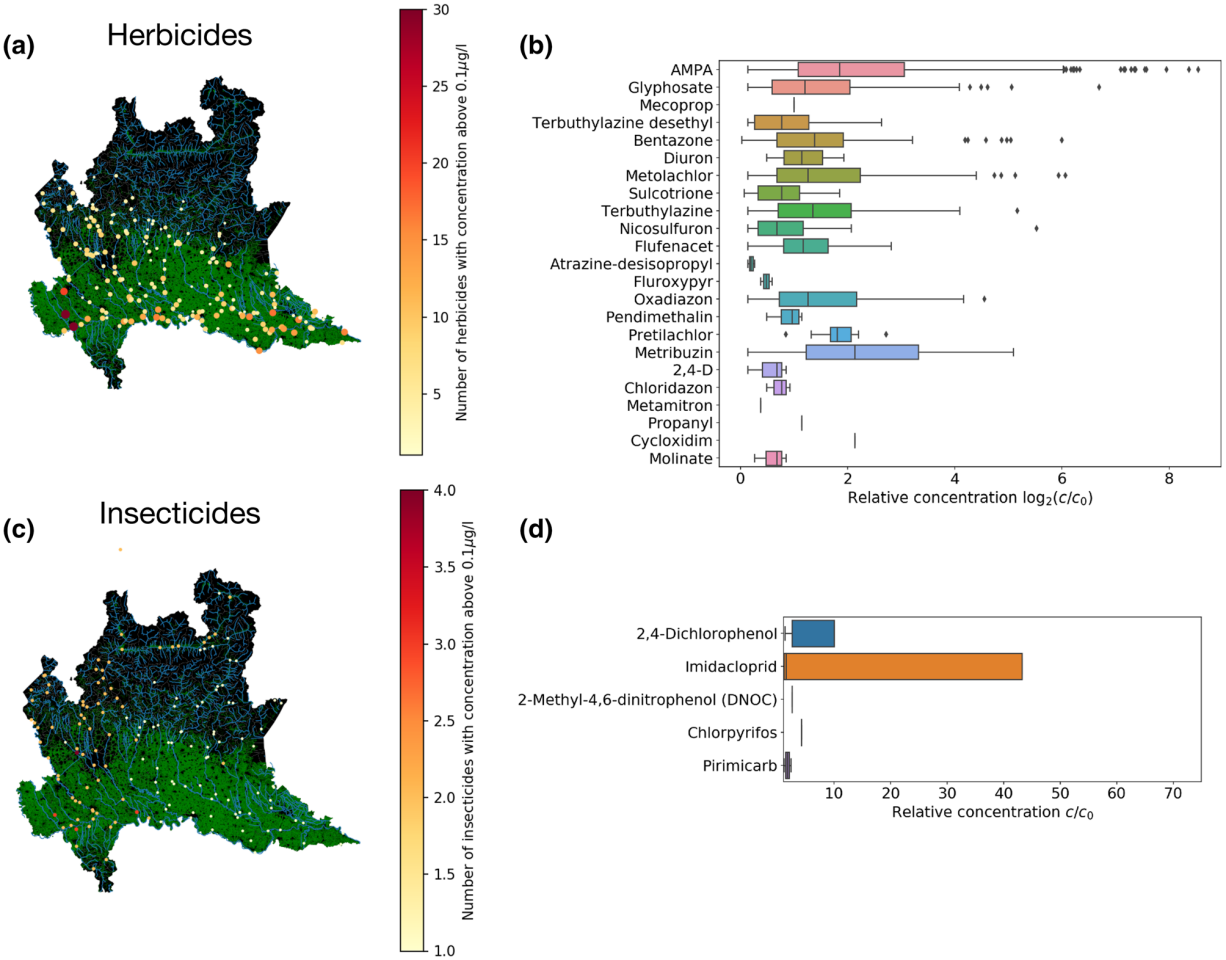
Distribution of herbicides and insecticides in surface waters. (**a**) Map showing where herbicides with $c>c_0=0.1$ μg/l have been recorded. The color code of the markers represents the number of substances found in the same location at the same time. Cultivated areas are colored in green, non cultivated areas are in black and rivers in cyan. (**b**) The distribution of relative concentrations $c/c_0$ of herbicides (only when $c>c_0$) Error bars in the boxplot are 1.5 of the inter-quartile range. (**c**) Same as (**a**) but for insecticides. (**d**) Same as (**b**) but for insecticides. The map was created in python3.7 using the geopandas (0.6.1) package (https://geopandas.org).

We first analyze the concentration and localization of individual substances. Figure 1a,c display the geographical distribution and the number of herbicides and insecticides detected in surface waters with a concentration larger than $c>c0=0.1$ μg/l (see “Materials and methods” for details). The points relative to the sampling stations have different sizes and colors: size and color gradient of each point represent the relative pesticide concentration and corresponds to the maximum concentration found for each location (for more details see “Materials and methods” section). The figure clearly shows that herbicides are detected less in the alpine area (the northern part of the map) while insecticides are equally distributed all over the region. However, insecticides are clearly detected close to valleys (such as Valtellina) where fruit trees and vineyards are cropped. We then report in Fig. 1b,d box plots of the concentrations—in units of $c_0$—of the substances with concentration exceeding $c_0$. The graphs show that a wide variety of pesticides with predominant presence in terms of concentrations of AMPA and Glyphosate among the herbicides, and Imidacloprin among insecticides. In particular AMPA can reach concentrations up to 20 μg/l which is 200 times the value of $c_0$. Similarly, Imidacloprin is found in concentrations that are up to 40 times larger than $c_0$.

**Figure 2:**
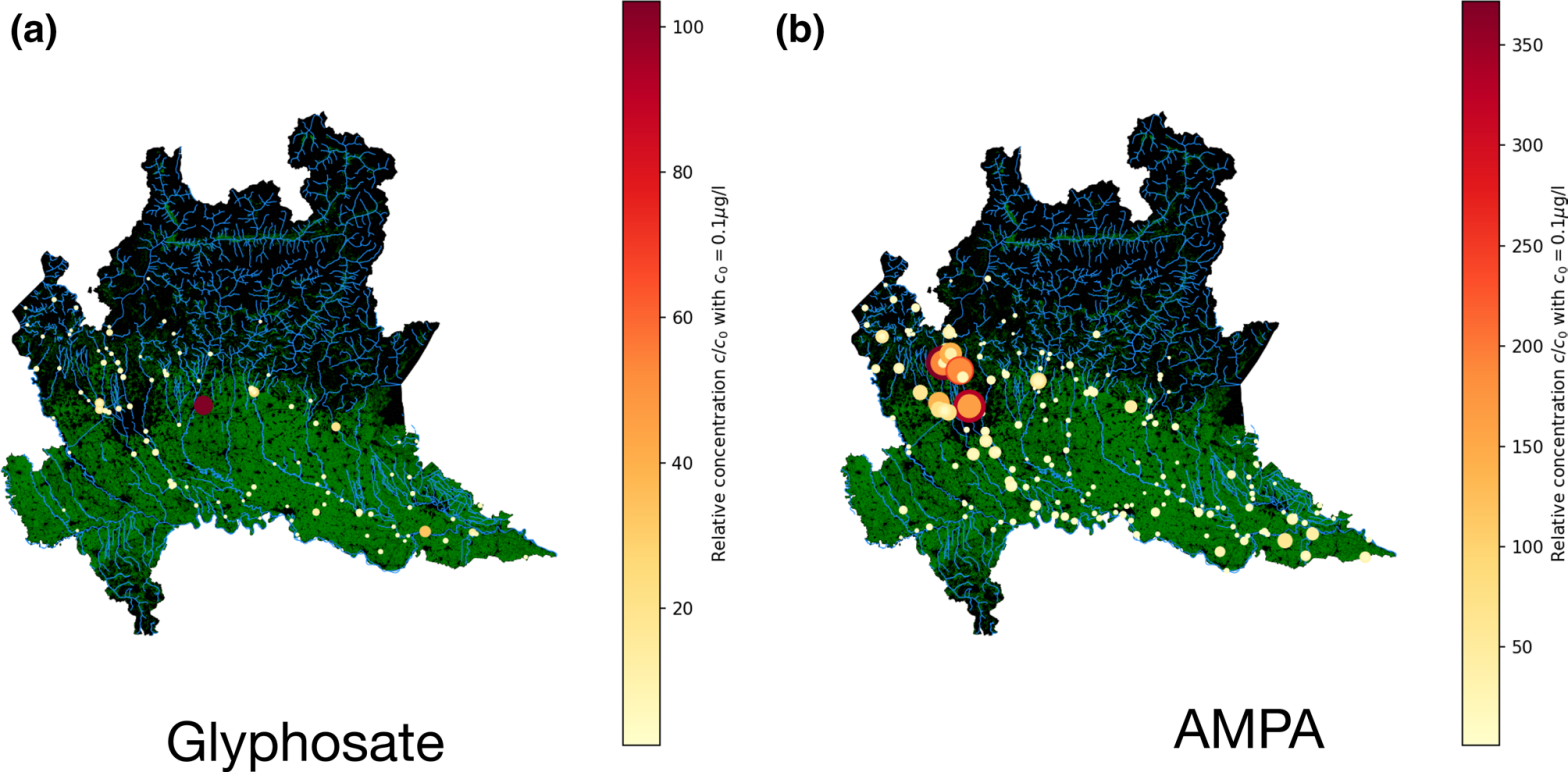
Glyphosate and AMPA in surface waters. Relative concentration of (**a**) glyphosate and (**b**) AMPA. We only report data when $c>0.1$ μg/l. Colorbar and marker size both indicate the relative concentration. Cultivated areas are colored in green and rivers in cyan. The map was created in python3.7 using the geopandas (0.6.1) package (https://geopandas.org).

The massive presence of AMPA and Glyphosate in surface waters is confirmed in Fig. 2 where we show the geolocalization and relative concentration of Glyphosate (Fig. 2a) and its metabolite AMPA (Fig. 2b). We observed nine records relating to Glyphosate exceeding the concentration of 10 μg/l while AMPA is found at a concentration of 134 μg/l. Figure 2 shows that these substances are both very abundant and widespread mostly in cultivated areas (in green in the map). In particular, it is interesting to notice the high levels of Glyphosate detected in the area of Roggia Vignola, an important irrigation channel near the town of Trevglio (see the large red dot in Fig. 2a). AMPA is accumulated in the North West area of Lombardy, which corresponds to the territory of the Varese province, where we find Olona, Seveso, Bozzente and Lura, four known polluted rivers^17, 18^.

**Figure 3:**
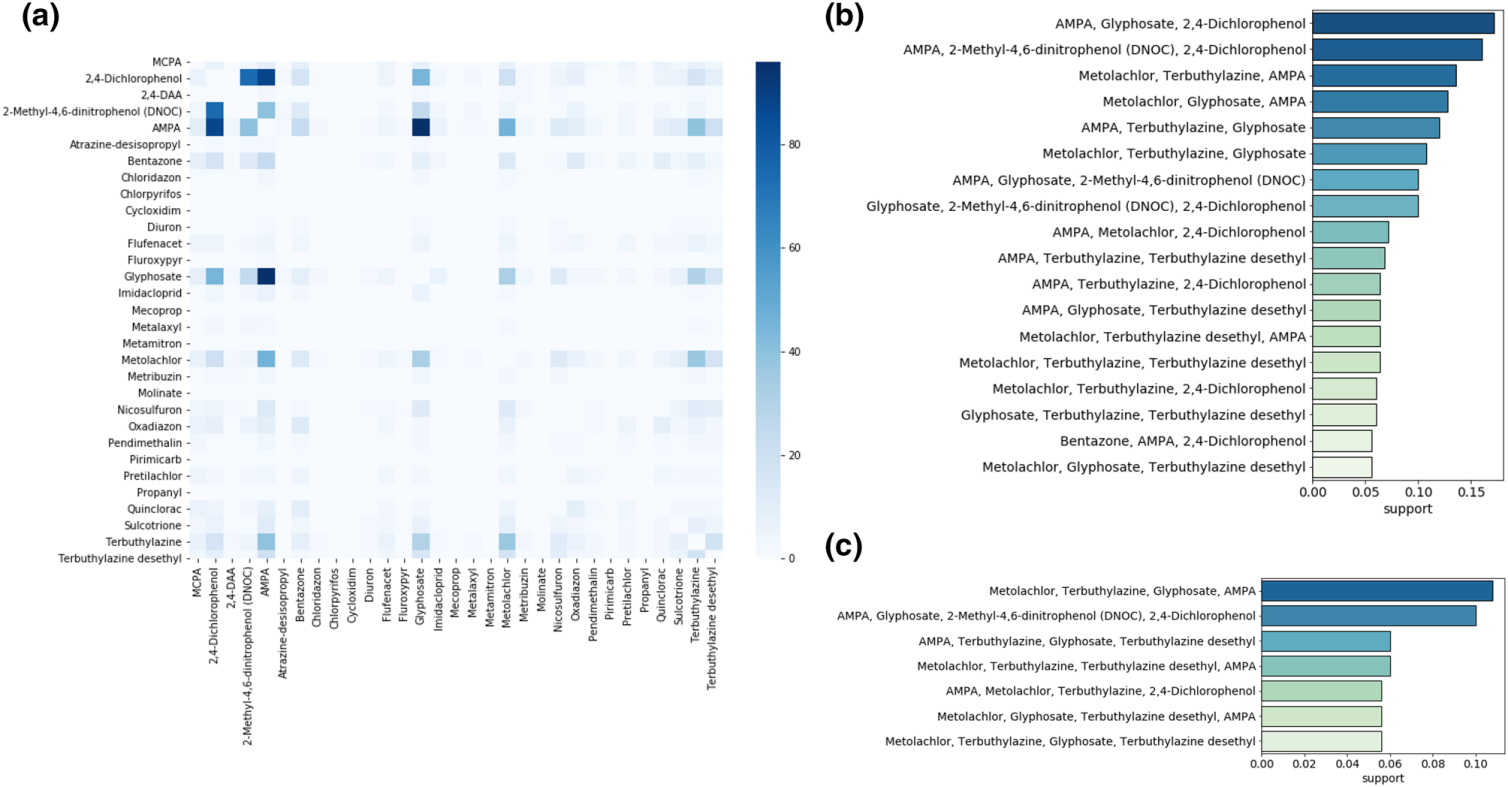
Clusters of substances in surface waters. (**a**) Substance cross-correlation matrix showing the number of times two substances (herbicides or insecticeds) have been found together in the same location and at the same time both with $c>c_0=0.1$ μg/l. The heatmap was created using python3.7 and the seaborn (0.11.0) package (https://seaborn.pydata.org/). (**b**) Fraction of instances in which three substances have been recorded (all with $c>c_0$) in the same location at the same time. (**c**) Fraction of instances in which four substances have been recorded (all with $c>c_0$ in the same location at the same time).

In order to identify which of the substances are co-present at high concentration in the same place, we constructed a cross-correlation matrix showing the number of times two substances, herbicides or insecticides, are recorded in the same location and at the same time both with $c>c_0$ (Fig. 3a). The matrix highlights that several substances are co-present, in particular AMPA and Glyphosate that are often found together but also in combination with other substances. To quantify multiple combinations of substances, we use the Apriori algorithm^12^ which is able to find association rules in a database. Our analysis shows that exist clusters of colocalized substances. of multiple substances are found in the same location. This result is summarized in Fig. S1, showing the distribution of cluster sizes found in surface waters and groundwater. The cluster sizes can be particularly large in surface waters, where we observe clusters composed by up to 17 substances. The majority of clusters, however, are composed by 4 substances or less. In groundwater, clusters are smaller and no cluster of size larger than 3 is ever observed.

To have a closer look at the composition of the most abundant clusters, we concentrate our analysis on clusters of size 4 or less. In Fig. 3b,c, we show the probabilities of finding clusters of three and four substances in the same location with $c>c_0$ in surface waters. We thus obtain a ranked list of the most likely clusters of substances. In particular, Glyphosate and AMPA are often found in combination with Terbuthylazine, Bentazon, 2,4-Dichlorophenol and Metolachlor.

**Figure 4:**
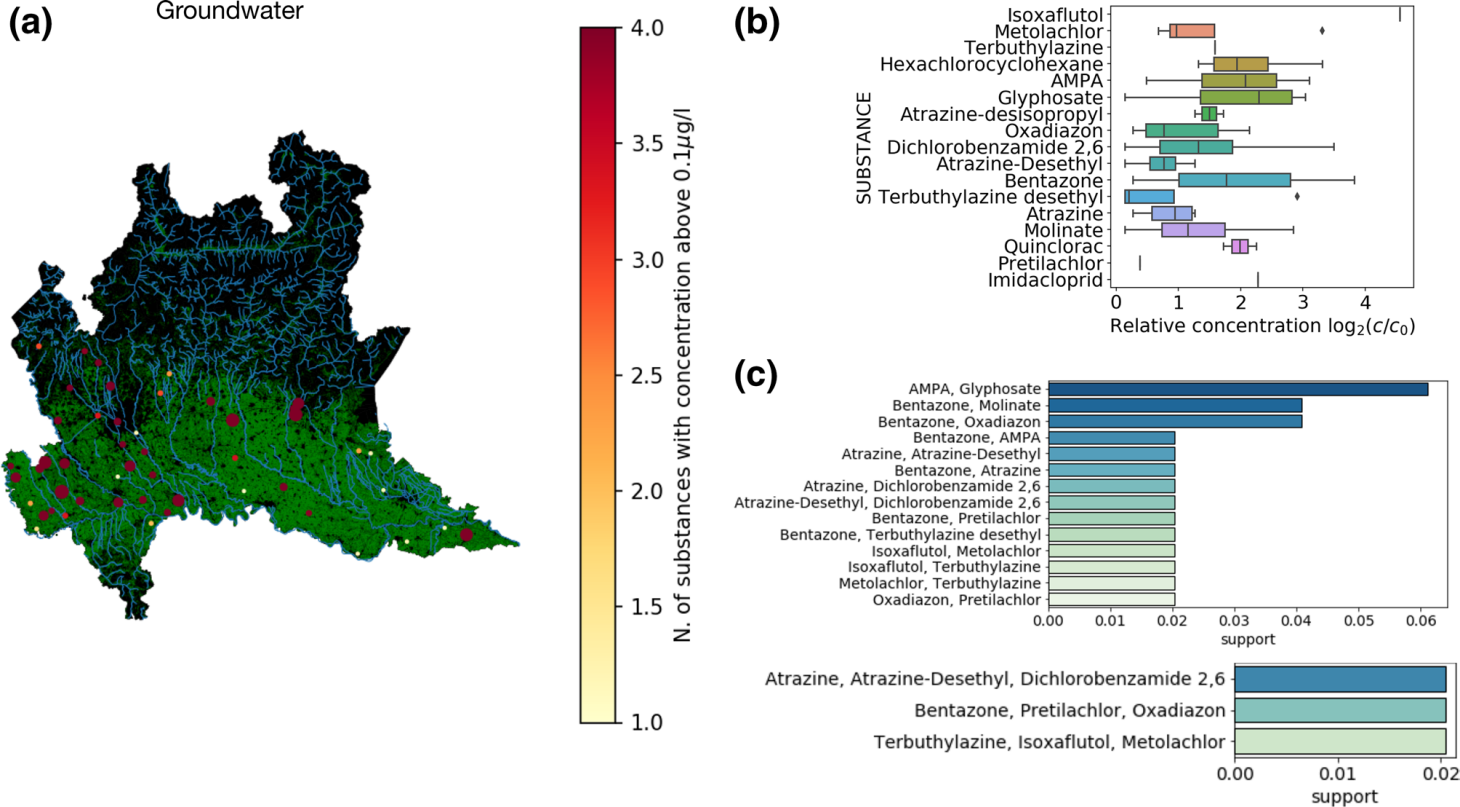
Clusters of substances in groundwater. (**a**) Map showing the sites where herbicides and insecticides have been found with $c>c_0=0.1$ μg/l. The color code of the marker represents the number of substances found in the same location at the same time. Cultivated areas are colored in green and rivers in cyan. (**b**) The distribution of relative concentrations $c/c_0$ (only when $c>c_0$). Error bars in the boxplot are 1.5 of the inter-quartile range. (**c**) Fraction of instances in which three substances have been recorded (all with $c>c_0$) in the same location at the same time. (**d**) Fraction of instances in which three substances have been recorded (all with $c>c_0$ in the same location at the same time). The map was created in python3.7 using the geopandas (0.6.1) package (https://geopandas.org).

We then analyzed groundwater with the same approach. As shown in Fig. 4a, the map shows the sites where herbicides and insecticides have been found with $c>c_0=0.1$ μg/l and the color code shows the number of substances found in the same location at the same time. The boxplots with the recorded concentrations are reported in Fig. 4b, showing that Glyphosate and AMPA are found at high concentrations, often two to four times the value of $c>c_0=0.1$ μg/l. We also find again the substances found in surface waters such as Terbuthylazine and Bentazon. We then perform again the clustering analysis with the apriori algorithm to identify groups of substances occurring together in the same location. Figure 4c,d report the clusters of two and three substances, showing again that similar patterns than those observed in surface waters. The difference is that the abundance of substances is smaller in groundwater than in surface waters, but despite this clusters of substances are still observed.

While the number of substances found in large concentration is large, there is a net prevalence of AMPA and Glyphosate both in surface and groundwater. Twelve herbicides and four stable metabolites are present in combination with Glyphosate and/or AMPA (Figs. 3, 4 and Table S3). Among these substances DNCO, Metolachlor, Molinate, Oxadiazon, Atrazine (Desethyl Atrazine), Dichlobenil (2,6 Dichlorobenzamide) were no longer in the market. We then chose for further analysis Bentazone and Terbuthylazine since they are always present in combination with Glyphosate and AMPA (Fig. 4b).

**Figure 5:**
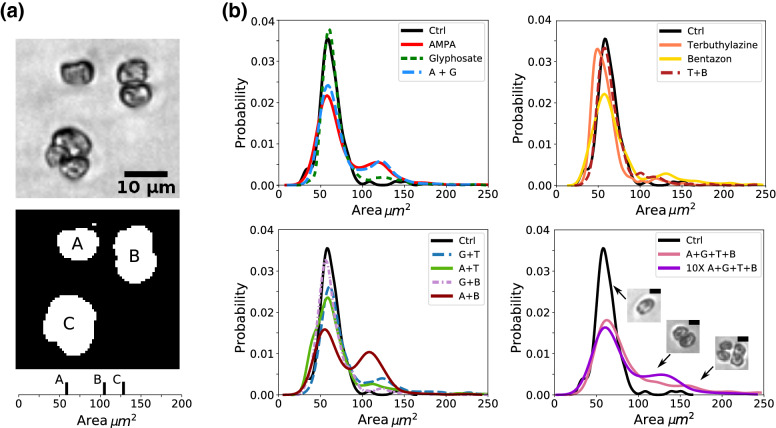
(**a**) Figure shows an exemplified image of *C. reinhardtii* single cell and aggregates of two and more cells. Binary masking was created in order to calculate the area of each cell/aggregate. Bottom panel shows the values obtained for the three particles shown. Note that area of the aggregate does not scale linearly with the number of particles due to three-dimensional conformation of the aggregates. (**b**) Size particle distribution obtained as described in (**a**) and in MM section for all the experimental conditions, as in legend. The presence of multiple peaks in the distributions suggests the presence of aggregates, expecially in presence of AMPA and AMPA in combination with Bentazon (A + B) compared to control condition. The presence of all the four substances at concentration equal to the one detected in surface waters (A + G + T + B) or ten times higher (10 × A + G + B + T) gives a broad distribution of sizes.

To investigate the possible synergistic impact of these substances on the environment, we used *C. reinhardtii* as a biological sensor. Using *C. reinhardtii* we quantified the possible effect of AMPA, Glyphosate, Bentazon and Terbuthylazine, individually or in combination, on the growth rate of the algae after 7 days and quantifying the presence of palmelloids. As shown in Fig. S2, we did not observe any significant effects on *C. reinhardtii* growth rate. However, we found that the exposure to AMPA and Glyphosate, alone or in combination with Terbuthylazine and Bentazon as well as the mix of the four compounds, leads to a broad size distribution with the presence of peaks in the larger sizes distribution, that is the typical dimension of cells aggregates (palmelloid shape) (Fig. 5).

## Discussion

Lombardy is the richest Italian region and one of the areas with the highest agricultural production rates, not only in Italy but in all Europe. Conventional agriculture spreads over 94.5% of Lombardy covering about one million hectares, while organic farming currently accounts only for 5.5% of the area. The intense exploitation of agricultural soils is also accompanied by a large use of pesticides. In all Europe, about 37 million tons of insecticides, 127 of herbicides and 168 of fungicides have been used in 2018, according to the Europaean Environmental Agency Environmental (EAE). Eurostat data from 2020 also reported that Italy ranks third among European countries, after Spain and France, for pesticide consumption. The analysis of data collected by the Regional Environmental Protection Agency (ARPA) in 2018 for surface and groundwater goes in the same direction, showing how herbicides and insecticides are important pollutants in Lombardy. Less pollutants above the legal threshold are present in groundwater as compared to surface water. In addition, the concentration of each compound is typically lower in groundwater than in surface waters. Another interesting aspect resulting from our analysis is that that herbicides are detected less in the alpine area while insecticides are present allover the region. Interestingly, insecticides have been detected in proximity to valleys, such as Valtellina, that are rich in fruit trees and vineyards. Therefore, we suggest that intense agricultural areas like Valtellina should be monitored in the future.

Among the herbicides present in both surface and groundwater above the legal threshold, we found compounds that are no longer in the market such as Molinate, Oxadiazon, Atrazine (Desethyl Atrazine), Dichlobenil (2,6 Dichlorobenzamide) and others that are still in the market such as AMPA, Glyphosate, Bentazon and Terbuthylazine.

Glyphosate is the world’s most widely used herbicide in agriculture since more than four decades. It is intensively applied to agricultural fields, before planting the crop, pre- or post-harvest, in both conventional and reduced/no-till farming, to control the growth of annual and perennial weeds. It is marketed as having no effect in animals since it is designed to specifically inhibit an enzymatic pathway required for protein synthesis unique to plants. In particular, Glyphosate is reported to be able to block plant growth by inhibiting the 5-enolpyruvylshikimate-3-phosphate synthase (EPSPS)^19, 20^. The EPSPS is a particular enzyme presents in plants, bacteria and fungi, but not in metazoans. Recently, Glyphosate was shown in Daphnia, a small planktonic crustaceans, to bind toxic metals present in the soil becoming more toxic on the crustaceous^21^.

Aminomethylphosphonic acid (AMPA) is one of the primary degradation products of Glyphosate. AMPA is normally present in soils and waters due to its chemical stability and it was found to be 3–6 times more resistant than Glyphosate against degradation^22^. There is no specific toxicity reported in the literature with the exception of a recent study showing the synergistic interaction between AMPA and another surfactant (polyethoxylated tallow amine, POEA) in affecting the development of zebrafish embryos (*Danio rerio*)^23^. The geolocalization analysis of AMPA showed that it is present in the agricultural areas of Lombardy. In particular, an accumulation of AMPA is visible in the North West area of Lombardy, which corresponds to the territory of the Varese Province, a well known polluted area^17, 18^. Although the toxicity of AMPA is still unclear, our pipeline offers a useful approach to monitor the possible impact on the territory of this pollutant as a function of time, in particular when it is present with others pollutants at high concentration.

Bentazon and Terbuthylazine, are important herbicides applied to maize and other crops to control pre-emergence or early post-emergence broadleaf and grass weeds. In the environment, they form metabolites such as N-methyl-bentazone, desethyl-Terbuthylazine, 2-hydroxy-Terbuthylazine^24, 25^. Bentazon and Terbuthylazine have a comparable mechanism of action. These two pesticides act as inhibitors of photosynthesis by blocking the electron transfer flow in photosystem II (PSII)^26^, the latter in turn may induce secondary effects in several metabolic pathways^27, 28^. These energized compounds can promote oxidative damage in chloroplasts proteins and membranes of photosynthetic cells^29^, causing cell death.

It is clear from the geolocalization analysis that the environment is exposed to multiple pollutants each at concentration above the legal threshold. The possible synergistic effect of the exposure to multiple pollutants is often neglected in the literature, concentrating mostly on the effect of a single pollutant. To investigate this point, suggesting a simple and easy pipeline, we decided to expose a bioindicator to the contaminants we observed in the waters.

Bioindicator organisms, such as algae, are widely used for water quality assessment but also to identify, qualify and quantify pollutants effects on environment^30^. *C. reinhardtii*, is a single-cell eukaryotic green alga found in temperate soil habitat, temporary pools, stagnant water and melting ice routinely used in the laboratory as a model organism for toxicological studies. This photosynthetic green alga is a robust model for plant cell and meets all the requirements of modern ecotoxicology for “good” test-systems: fast growth rate at the lab scale^31^, relatively inexpensive^32^, rapid responses to changes in the environment in which it resides^33, 34^, good resolution, high sensitivity^34^ and easily genetically modifiable. For these reasons, it is know as “the green yeast”^35^. *C. reinhardtii* has been exstensively used to study responses to various abiotic stress agents such as osmolytes, temperature, nutrient starvation, heavy metals and oxidative stress^36, 37^. Several studies reported that *C. reinhardtii* cell viability was found to be inversely proportional to the stress dose^33^ and duration of exposure to stressors. Moreover, while in physiological conditions *C. reinhardtii* appears as a single swimming cell thanks to the presence of its flagella, in stress conditions immediately reacts either with flagella paralysis or loss^38^ and, as later response, with the formation of clumps of non-motile cells called ‘palmelloids’^39^. The transition from the unicellular life to multicellularity is a macroscopic morphological stress response that can be used to measure the global stress effect on the algae, even at low concentrations of stressors. Finally, *C. reinhardtii* plays an important role in the equilibrium of aquatic ecosystems, representing the first level of the trophic chain. Due to their highly sensitive nature, microalgae are claimed as early indicator of deteriorating environmental conditions^40^.

In the present study, we exposed *C. reinhardtii* to a mix containing the higher concentration of AMPA, Glyphosate, Bentazone or Terbuthylazine found in surface waters. While the impact on the growth of *C. reinhardtii* was not significant^9–11^, we found a significant increase of palmelloids, which represents a stress response of algae to environmental factors after 7 days of exposure. All together our findings based on a combination of data analysis and experiments highlight the potential environmental relevance of synergistic effects of multiple pollutants accumulating in surface and groundwater. Finally, since the proposed pipeline is able to provide results in a short time and with reduced costs, and it is potentially applicable to any other Italian and foreign territory, its use as a new testing routine could be relevant for a more efficient environmental monitoring.

## Supplementary Information

Supplementary Information 1.

Supplementary Table S1.

Supplementary Table S2.

Supplementary Table S3.
