# Supplementary material for: Synergistic effects of contaminants in Lombardy waters
Source: Sci Rep. 2021 Jul 6;11:13888. doi: 10.1038/s41598-021-93321-6 (PMC8260714; doi:10.1038/s41598-021-93321-6)
Supplement: Supplementary file 1 — Supplementary Information 1. [file 41598_2021_93321_MOESM1_ESM.pdf]

SI for: Synergistic effects of contaminants in Lombardy waters

Caterina AM La Porta[a,b,c,f], Maria Rita Fumagalli[a,b,c], Stefano Gomarasca[b], Maria Chiara Lionetti[a,b], Stefano Zapperi[a,d,e], Stefano Bocchi[b]

[a] *Center for Complexity and Biosystems, University of Milan, via Celoria 16, 20133 Milano, Italy*
[b] *Department of Environmental Science and Policy, University of Milan, via Celoria 26, 20133 Milano, Italy*
[c] *CNR - Consiglio Nazionale delle Ricerche, Istituto di Biofisica, via Via De Marini 6,16149 Genova, Italy*
[d] *Department of Physics, University of Milan, Via Celoria 16, 20133 Milano, Italy*
[e] *CNR - Consiglio Nazionale delle Ricerche, Istituto di Chimica della Materia Condensata e di Tecnologie per l'Energia, Via R. Cozzi 53, 20125 Milano, Italy*
[f] *Corresponding author: caterina.laporta@unimi.it*

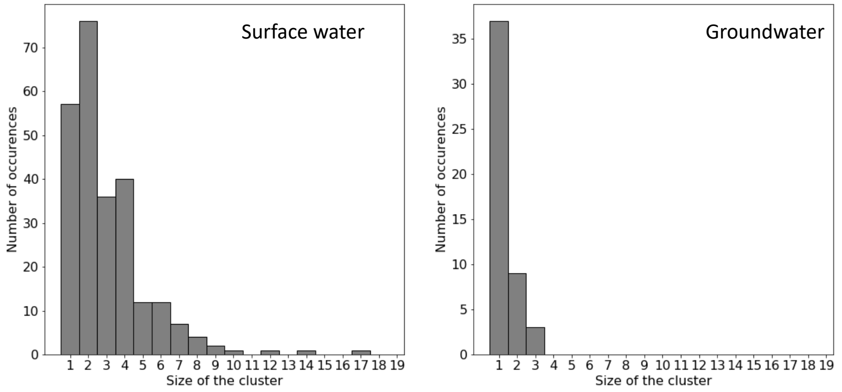

Figure S1: **Cluster size distributions.** The histograms report the number of instances in which a cluster of substances was found in the same location for surface water (left) and groundwater (right). In surface waters, we observe clusters of up to 17 different substances, with the majority of clusters of size up to four substances. No cluster with more than 3 substances is ever observed in groundwater.

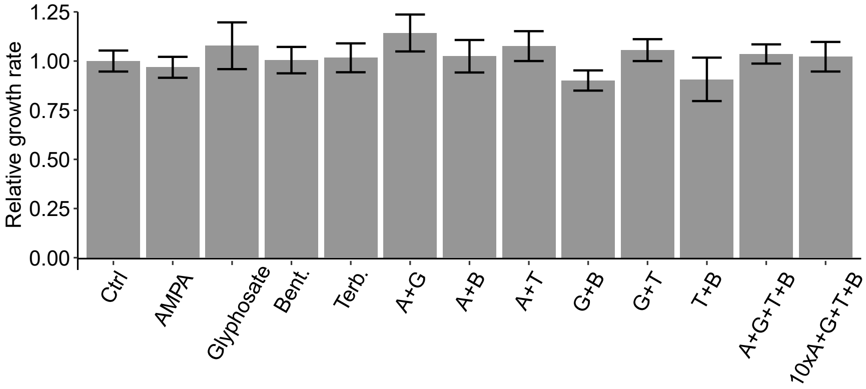

Figure S2: *C.reinhardtii* **relative growth rate** *C. reinhardtii* cells were cultured in TAP medium containing glyphosate, AMPA, bentazon (Bent.), terbuthylazine (Terb.) separately and in combination at the same concentrations detected in shallow water. Additionally, we also considered a concentration ten times higher for the mix of all the four substances (10X A+G+B+T), and cells grown in TAP medium without any other contaminant were used as control (Ctrl) as describer in Materials and Methods section. *C. reinhardtii* growth was monitored by measuring optical density at 680 nm of 100 μL cell culture using a microplate reader (Ensight, Perkin Elmer). Growth rate was calculated for every experimental condition considered as the log2 ratio between optical density immediately after the seeding and after seven days. Plot shows the average growth rate relative to control condition over four to five (Ctrl,AMPA, bent., A+B, A+G+B+T,10X A+G+B+T) experimental replica. For each replica and condition, two independent measurements were performed. Error bars are standard errors of the mean. No statistically significant differences were found using Welch Two Sample t-test between all the conditions.

Supplementary table captions

Table S1: List of substances (herbicides and insecticides) analyzed in the paper for surface and groundwater.

Table S2: Classes of substances and quantities of products measured by ARPA in the Lombardy surface and groundwater in 2018.

Table S3: W-T: water tipology; RE-LI: revoked license ; YE-RE: year of last revoked; AU-PR: authorized products; RE-PR: re-registered products; EX PR: expired license; surface*, under*: stable products derived from degradation of Glyphosate (AMPA), Dichlobenil (2,6,Dichlorobenzammide) Atrazine (Desethyl Atrazine), Terbuthylazine (Desethyl Terbuthylazine)
